# A highly active worm-like PtMo nanowire for the selective synthesis of dibenzylamines†

**DOI:** 10.1039/c8ra00787j

**Published:** 2018-02-26

**Authors:** Shuanglong Lu, Pengyao Xu, Xueqin Cao, Hongwei Gu

**Affiliations:** Key Laboratory of Organic Synthesis of Jiangsu Province, College of Chemistry, Chemical Engineering and Materials Science, Collaborative Innovation Center of Suzhou Nano Science and Technology, Soochow University Suzhou China 215123 hongwei@suda.edu.cn

## Abstract

Worm-like nanowires are among the most active nanomaterials. In this study, we report the synthesis of dibenzylamine (DBA) motifs from reductive amination of either aldehydes or nitriles catalyzed by entirely new worm-like PtMo nanowires (PtMo WNWs). Under the assistance of H_2_ gas, PtMo WNWs can be prepared in a facile manner, following which, their structure and composition are characterized by TEM, XRD, XPS, *etc.* Upon careful optimization of reaction parameters, the as-prepared PtMo WNWs work effectively in the activation of dihydrogen molecules, and both aldehydes and nitriles can be used as starting materials to fabricate DBAs under mild and green conditions. The reaction kinetics has been investigated, which reveals that the PtMo WNWs show superior activity in the conversion of imines into amines. This study provides a practical advancement in the preparation of amines. Moreover, the protocol reported herein is feasible for the synthesis of worm-like nanostructures with designed composition for various catalytic applications.

## Abstract 2

An efficient synthetic pathway of dibenzylamine (DBA) motifs from reductive amination of either aldehydes or nitriles catalyzed by highly active worm-like PtMo nanowires.

## Graphical abstract

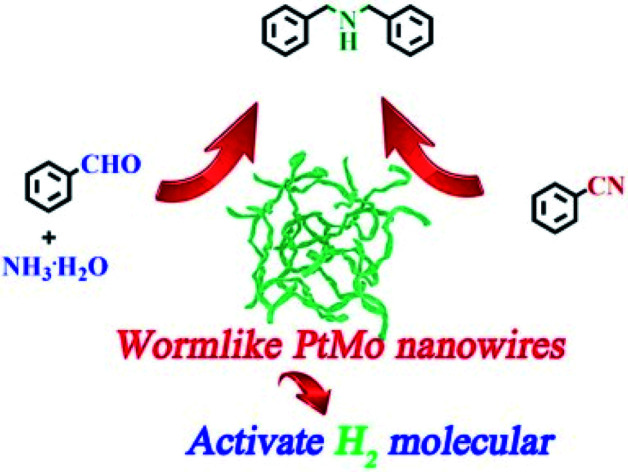

## 1. Introduction

Dibenzylamine (DBA) motifs are important compounds with extensive applications in rubber compounds, fine chemicals, corrosion inhibitors, and drug formulations;^1–4^ in this regard, the development of methods allowing the selective synthesis of DBA is of great significance in organic chemistry. Through significant research efforts made in this direction, synthetic routes, such as direct base-promoted mono-*N*-alkylation^5^ or alkylative amination,^6,7^ have been developed for the synthesis of DBA motifs. However, problems such as the use of expensive starting materials, tedious workup procedures, low selectivity, and formation of large amounts of wasteful salts still remain to be addressed.

Reductive amination is a highly versatile and robust method for various transformations involved in the C–N bond construction.^8–10^ It offers compelling advantages such as mild reaction conditions, inexpensive reagents, and a wide availability of substrates over other classical amine synthesis methods.^11–13^ Among various reduction amination processes, the activation of dihydrogen and the catalytic reduction of unsaturated organic molecules is a fundamentally promising green process for the production of amines.^14–18^ Conventional strategies, using Raney® Ni or Tin compounds, suffer from low selectivity and poor stability.^19^ Alternatively, transition metal-based organometallic complexes have been proven to be efficient catalysts to cleave dihydrogen molecules,^20^ but their complete removal from the reaction product presents a big concern when it comes to the production of pharmaceutical intermediates. Moreover, precise design and synthesis are required for organometallic catalysts. Recently, use of well-defined metal nanostructures in catalytic hydrogenation processes has become a rapidly growing area of research.^21–25^ Somorjai and Yang reported the catalytic hydrogenation of pyrrole using platinum nanocrystals and found that the activity and selectivity were heavily dependent on the size and shape of the nanocrystals.^26^ Cao and his coworkers utilized gold-based nanostructures to activate dihydrogen molecules.^27^ Unsaturated organic compounds, such as quinolones, were hydrogenated under mild conditions.

**Scheme 1:**
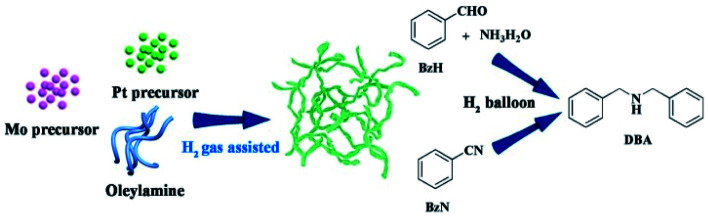
Schematic of the synthesis of PtMo WNWs and its catalytic application in the DBA synthesis from reductive amination of either BzH or BzN.

Herein, we report entirely new worm-like PtMo nanowires (WNWs) prepared through facile co-reduction of Pt and Mo precursors under the assistance of H_2_ gas. The as-prepared nanowires work effectively in the activation of dihydrogen, following which, DBAs can be obtained from the reductive amination of either benzaldehyde (BzH) or benzonitriles (BzN) under mild and green reaction conditions (Scheme 1). Worm-like nanowires are among the most active nanostructures, which are widely studied in both electro-catalysis and organic transformation.^28–30^ The wavy structure and the incorporation of Mo are favorable to enhance the catalytic activity and durability, respectively.^31^ Furthermore, the newly developed strategy for PtMo WNWs simplifies the synthetic process for nanostructured catalysts, providing a much more practical pathway for catalytic applications.

## 2. Experimental

### Synthesis of worm-like PtMo nanowires

To prepare worm-like PtMo wavy nanowires, a Pt precursor (platinum acetylacetonate, 0.125 mmol, 49.2 mg) and a Mo precursor (molybdenyl acetylacetonate, 0.125 mmol, 40.8 mg) were added to OAm (31 mmol, 10 mL) in a three-necked flask, followed by stirring for 30 min at 100 °C under a N_2_ atmosphere to ensure that the precursors were dissolved thoroughly. The solution was cooled down to room temperature and transferred into an autoclave, which was then charged with H_2_ to 5 bar. The autoclave was heated to 165 °C and kept at this temperature for 2 h under stirring. The resulting black colloidal products were obtained by centrifugation and washed several times with ethanol and chloroform.

### Material characterization

The morphologies of the worm-like PtMo nanowires were determined by a TEM (Tecnai G220, FEI, USA) equipped with a Gatan CCD794 camera operated at 200 kV. The HAADF-STEM, elemental mapping, HR-TEM, and EDX of a single nanohybrid were carried out using a Tecnai G2 F20 instrument at an accelerating voltage of 200 kV. The XRD pattern was obtained using an X'Pert-Pro MPD diffractometer (the Netherlands PANalytical) with a Cu Kα X-ray source (*λ* = 1.540598 Å).

### General procedure for the reductive amination of aldehydes and nitriles

Reactants, solvents, and catalysts were added to the reaction tube that was then sealed. The reaction tube was evacuated thrice and then flushed with hydrogen at a certain temperature under an atmosphere of hydrogen. The resulting product mixtures were analyzed by GC (VARIAN CP-3800 GC, HP-5 capillary column, FID detector) and GC-MS (VARIAN 450-GC & VARIAN 240-GC) equipped with a CP8944 capillary column (30 m × 0.25 mm). Products were purified by flash chromatography and characterized by ^1^H NMR and ^13^C NMR spectroscopies.

## 3. Results and discussion

The PtMo WNWs were prepared through co-reduction of Pt and Mo metallic precursors in oleylamine (OAm) by heating to 165 °C in an autoclave under H_2_ gas. The detailed procedure is provided in the experimental section.

**Fig. 1:**
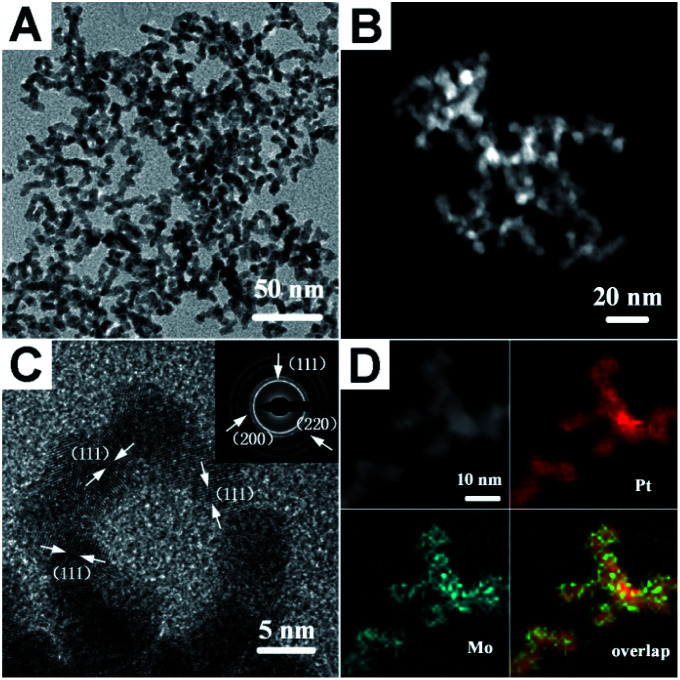
(A) Representative TEM image; (B) HAADF-STEM image; (C) HRTEM image, the inset shows the SAED pattern; and (D) high-magnification HAADF-STEM image and the corresponding elemental mapping of the worm-like PtMo nanowires.

Fig. 1A and B show the representative transmission electron microscopy (TEM) and high-angle annular dark-field scanning transmission electron microscopy (HAADF-STEM) images of the as-prepared PtMo WNWs, respectively, that are highly uniform with nearly 100% nanowire morphology. As can be seen, these nanowires are highly dispersed with a narrow size distribution. The average diameter of the product is around 4.8 nm, and the length is 100 nm. Impressively, the high-resolution TEM (HR-TEM) image shown in Fig. 1C reveals that these nanowires are composed of multiple crystalline domains, and the observed fringes are from the {111} planes. The continuous lattice fringes from the inner to the outer areas of PtMo WNWs without an obvious phase segregation demonstrate high amalgamation between Pt and Mo atoms. Moreover, the selected-area electron diffraction (SEAD) pattern shows a typical metallic face-centered cubic (fcc) structure, which is in agreement with the powder X-ray diffraction (PXRD) pattern (Fig. S1†). Intriguingly, there are no single peaks for Mo or Pt in the XRD patterns; this implies the purity of the PtMo alloy without undesired phases. The atomic ratio of Pt/Mo is 93.56/6.43, determined by an energy dispersive spectrometer (EDS) (Fig. S2†). The observed low content of Mo is due to the high stability of Mo precursors against inferior reductants, such as H_2_, resulting in incomplete reduction of Mo precursors, which is consistent with previous studies.^31,32^ The binding energy of Pt 4f is around 71.3, which is determined by the XPS analysis shown in Fig. S3,† and it shifts to a higher value relative to that of pure Pt NCs (70.9) due to the alteration of the Pt electronic structure with Mo atoms that downshifts the d-band center of Pt. The formation of the PtMo alloy is further investigated by the elemental mapping analysis (Fig. 1D). This reveals that Pt and Mo are well-distributed throughout the interior and exterior domains.

To demonstrate the high catalytic activity of these PtMo WNWs in the preparation of DBAs, BzH was selected as the substrate for the optimization of reaction conditions. Table 1 shows the reductive amination of BzH with aqueous ammonia as the economical N source.

**Table 1:** Optimization of reaction conditions using benzaldehyde as a substrate^a^

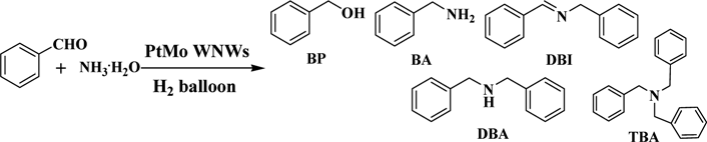							
Entry	Solvent	*T*/°C	Conv.^b^ (%)	Yield^b^ (%)			
				BP	DBI	DBA	TBA
1	Methanol	60	100	3.2	19.1	70.2	—
2	*n*-Heptane	80	100	3.1	—	91.4	5.5
3	Water	80	100	66.1	3.6	30.3	—
4	Ethanol	80	98.8	26.2	3.2	40.9	28.6
5	DMF	80	96.4	4.2	58.5	33.7	—
6	1,4-Dioxane	100	64.8	54.2	1.2	4.5	4.9
7	Toluene	100	100	16.7	—	—	83.3
8	*p*-Xylene	100	100	3.1	—	92.8	1.7
9	*o*-Xylene	100	100	2.4	—	92.4	2.8
10	*m*-Xylene	100	100	0.5	0.8	96.1	0.8
11	*m*-Xylene	80	100	16.6	10.1	67.0	—
12	*m*-Xylene	60	95.2	17.5	57.4	18.3	—
13	*m*-Xylene	40	97.2	9.4	74.1	13.8	—

a Reaction conditions: BzH (1.0 mmol), 25% aqueous ammonia (2 equiv.), and solvent (2 mL) at 1 bar H_2_ with 0.5 mol% PtMo WNWs for 24 h.

b GC yield.

Through GC-MS analysis, we have obtained the idea that besides DBA, side products, such as benzylalcohol (BP) benzylamine (BA), dibenzylimine (DBI), and tribenzylamine (TBA), can be detected (Fig. S4†). Various solvents were first screened at the corresponding temperatures by employing a H_2_ gas balloon (Table 1, entries 1–10). Polar solvents (entry 1, 3–6) seem to be unsuitable to improve the selectivity in the reductive amination of BzH *via* the PtMo catalysis. When *n*-heptane was used as a solvent (entry 2), the yield of DBA increased to 91.4%, a relatively satisfactory result. Interestingly, we obtained TBA as the main product when toluene was used (entry 7). The best solvent in our screening was found to be xylene, and the yields of 92.4%, 96.1%, and 92.8% were obtained for DBA in *o*, *m*, and *p*-xylene, respectively (entry 8–10). Based on these results, *m*-xylene was chosen as the best solvent for the following investigation on the temperature influence. Obviously, with a decrease in the reaction temperature, a corresponding steady decrease in the BDA yield was found, as presented in Table 1 (entry 10–13). The molar ratio between BzH and ammonia was also vital to the reduction activity. When ammonia was absent, BP was the major product. A higher or lower concentration of ammonia was more or less not favorable for DBA formation (Table S1†). All these observations indicate that PtMo WNWs present a good result for DBA synthesis.

Due to the highly efficient catalytic performance of PtMo WNWs in the reductive animation of the aldehyde under H_2_ gas, we examined the catalytic capability of PtMo in the reductive hydrogenation of nitriles (Table 2). Nitriles, which are bioavailable in nature, are a promising feasible alternative to fabricate DBAs because no additional N sources are required.^33,34^ However, how to enhance the selectivity for the reduction of nitriles still remains a challenge.^35–37^

**Table 2:** Optimization of the reaction conditions using benzonitrile as a substrate^a^

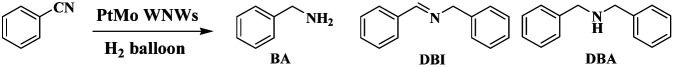						
Entry	Solvent	*T*/°C	Conv.^b^ (%)	Conv.^b^ (%)		
				BA	DBA	DBI
1	Methanol	40	94.9	—	91.0	1.5
2	*n*-Heptane	80	80.6	—	56.8	23.6
3	1,4-Dioxane	100	93.1	4.5	84.5	7.4
4	Toluene	100	98.2	2.3	91.0	4.9
5	Water	100	98.4	4.5	85.0	—
6	*p*-Xylene	100	98.5	3.1	91.3	—
7	*m*-Xylene	100	100	1.4	93.5	5.1
8	*o*-Xylene	100	100	—	92.4	7.6
9	Ethanol	40	98.0	4.0	86.3	7.7
10	Ethanol	60	100	1.5	94.3	4.2
11	Ethanol	80	100	—	95.4	4.6

a Reaction conditions: BzN (1.0 mmol) and solvent (2 mL) at 1 bar H_2_ with 0.5 mol% PtMo WNWs for 24 h.

b GC yield.

As indicated in Table 2, contrary to the situation when BzH was used, except heptane, most solvents were suitable for the reductive amination of BzN (Table 2, entries 1–9). Among them, ethanol was found to be the best solvent, yielding 95.4% DBA at 80 °C (Fig. S5†) after we carefully evaluated the temperature influence. In the control experiment, no reduced products were observed in the absence of either a catalyst or H_2_ gas (Table S2,† entry 1, 2). Moreover, commercial Pt/C (20%) was used in the reductive amination of BzH or BzN for comparison (Table S2,† entry 3, 4). Obviously, low selectivity was observed, and TBA emerged as the main side product. Pt nanoparticles and Pt nanorods with a smooth surface were also synthesized and applied in the reduction of nitriles for comparison (Table S3†). Apparently, worm-like PtMo nanowires show much better performance. The enhancement in the catalytic activity could be attributed to their wavy structures, which could provide numerous structural defects and grain boundaries. It has been previously suggested that the surface defects can serve as possible channels for incorporating small molecules into the surface region.^38–40^ The incorporation of Mo into Pt enhances the activity and stability of Pt, which has been comprehensively investigated in previous studies.^41^ Moreover, the synergistic electronic effect between two metals may have important role in the catalytic reaction.^42^ Hydrogen on the surface of Pt sites can migrate over to the Mo species, liberating the Pt active sites.^31^

**Fig. 2:**
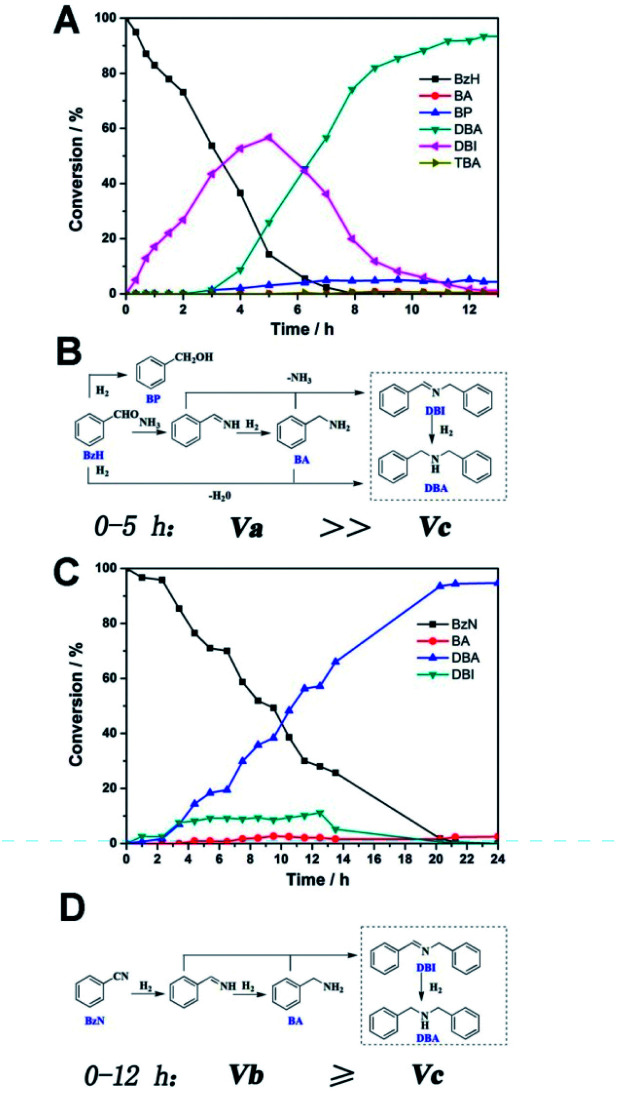
(A) and (B) Time–conversion plot and proposed mechanism for the DBA formation using BzH as the starting reactant under the optimized reaction conditions; (C) and (D) with BzN as the starting reactant.

Real-time GC measurement was used to track the kinetic processes of the transformation. Fig. 2A and C show the time–conversion plots when BzH and BzN are used as the starting reactants, respectively. As can be seen, with the consumption of the either starting substrate, the only intermediate that can be detected in common is DBI. However, they differ from each other in terms of the concentration of the DBI formed. For reduction amination using BzH, the concentration of DBI reached a peak value of 56.7% after 5 hours, whereas the amount of DBI, as shown in Fig. 2C, for BzN time–conversion remained stable at a steady level below 10.0%.

Based on these observations and our previous studies, we have proposed mechanisms for the two separate systems (Fig. 2B and D). We defined the reaction velocity of BzH to DBI as *V*_a_, BzN to DBI as *V*_b_, and DBI to DBA as *V*_c_. Apparently, as shown in Fig. 2A, the intermediate DBI formed cannot be successively consumed to form DBA; this leads to the accumulation of DBI in the mixture. *V*_a_ is far greater than *V*_c_.

It is reasonable that the two proposed pathways are available for BzH to form DBI, which are coupling of BA with phenylmethanimine or BzH itself (Fig. 2B). On the other hand, for BzN, only one pathway is available, and most of the time, the formation and consumption of BDI reaches a dynamic equilibrium; *V*_a_ is slightly greater or equal to *V*_c_. Moreover, trace amounts of side products, such as BA, BP, and TBA, can be detected in the BzH system, whereas only BA can be detected in the BzN system. Compared with our previous studies,^21,22^ it is highly noteworthy that *V*_c_ is significantly improved; this demonstrates that PtMo WNWs are much more efficient in the conversion of imines into amines. The potential application of PtMo WNWs is ongoing in our group, and we will communicate these results in due course.

To determine the scope of these two pathways in the synthesis of DBA motifs, typical substrates of aldehyde and nitrile were chosen to be subjected to their optimized reaction conditions. As shown in Table 3, irrespective of the electronic nature of the substituent, good to excellent yields of DBAs can be obtained from the reductive amination of either BzH or BzN (entry 1–4). Upon comparing *ortho*-, *meta*-, and *para*-substituted BzH or BzH (entry 1, 5 and 6), steric hindrance effect was proven to exert little influence on the reaction yield. Notably, the use of higher pressures led to an increase in the yield of DBAs and shortened the reaction time for some substrates (entry 2B).

**Table 3:** Formation of DBA motifs with different substrates through various pathways^a^^,^^b^

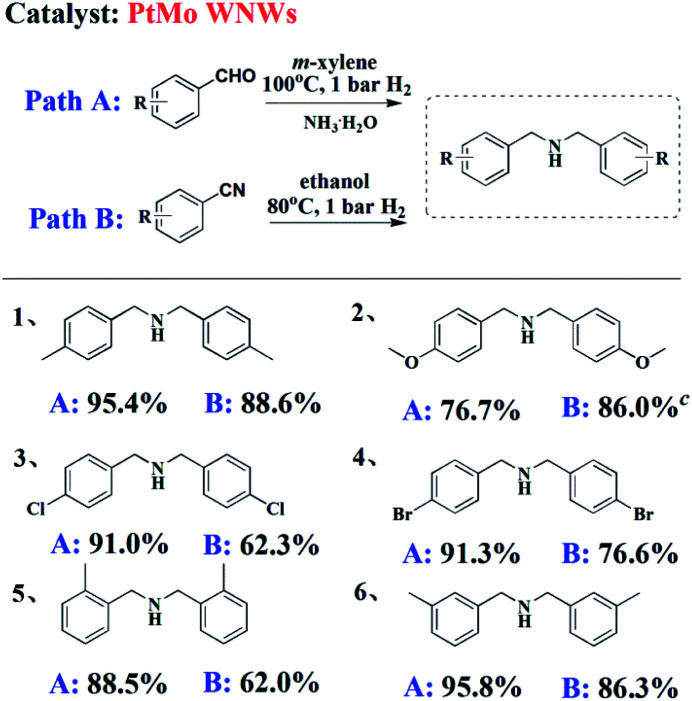

a Reaction conditions for Path A: BzH (1.0 mmol), 25% aqueous ammonia (2 equiv.), *m*-xylene (2 mL), 1 bar H_2_, 100 °C, 0.5 mol% PtMo WNWs for 24 h; for Path B: BzN (1.0 mmol), ethanol (2 mL), 1 bar H_2_, 80 °C, 0.5 mol% PtMo WNWs for 24 h.

b GC yield.

c 5 bar H_2_ for 8 h.

**Fig. 3:**
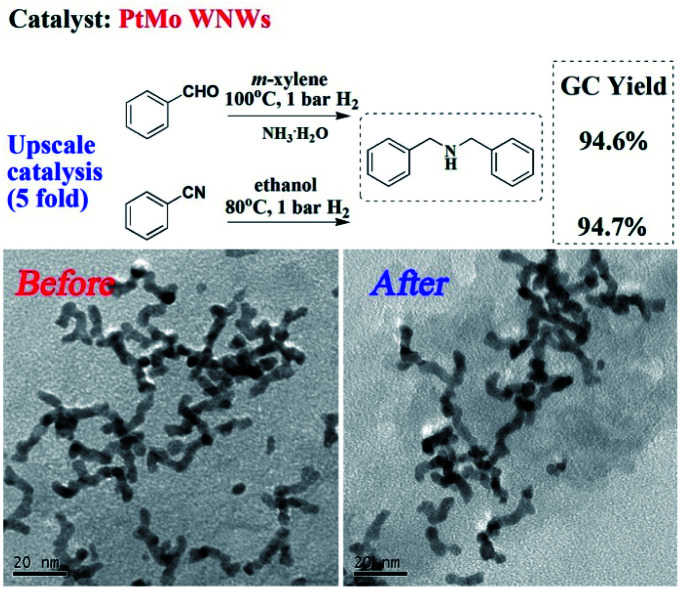
Upscale catalysis performance and recycling of the catalyst.

Finally, we investigated the upscale catalysis performance and recyclability of PtMo WNWs. As shown in Fig. 3, a yield of 94.6% for DBA was obtained when 5-fold BzH was subjected to the optimized reaction conditions. An equally satisfactory result was also found in the situation of BzN. The PtMo WNWs before and after the reaction were characterized by TEM images, as shown in Fig. 3. No significant changes were observed. Impressively, there was also no discernible loss in activity after several runs (Fig. S6†).

The excellent catalytic activity and stability of worm-like PtMo nanowires can be attributed to the following three factors: (1) the worm-like structure provides numerous catalytic sites as compared to a smooth structure. (2) The incorporation of Mo into Pt can enhance the activity and stability of Pt, which has been comprehensively investigated in previous studies.^41^ Moreover, the synergistic electronic effect between two metals may have an important role in the catalytic reaction.^42^ Hydrogen on the surface of Pt sites can migrate over to the Mo species, liberating the Pt active sites.^31^ (3) The nanowire network is less susceptible to aggregation and dissolution.

## 4. Conclusions

In conclusion, a facile method was developed for the preparation of entirely new PtMo WNWs. The as-obtained worm-like nanowires work effectively in the activation of dihydrogen, through which, DBAs are successfully synthesized by the reductive amination of either BzH or BzN. After careful evaluation of the kinetic processes, we found that the catalyst was much more active in the conversion of imines into amines due to its composition and morphological effects. Moreover, it can be readily recovered, and the catalytic system can be easily scaled up. Further study of this catalytic process and potential applications of PtMo WNWs are under investigation in our laboratory.

## Conflicts of interest

There are no conflicts to declare.

## Supplementary Material

RA-008-C8RA00787J-s001
