# Supplementary material for: A highly active worm-like PtMo nanowire for the selective synthesis of dibenzylamines
Source: RSC Adv. 2018 Feb 26;8(16):8755–60. doi: 10.1039/c8ra00787j (PMC9078585; doi:10.1039/c8ra00787j)

Electronic Supplementary Material (ESI) for RSC Advances.
This journal is © The Royal Society of Chemistry 2018

Supporting information

Highly Active wormlike PtMo Nanowire in the Selective Synthesis of Dibenzylamines

*Shuanglong Lu, Pengyao Xu, Xueqin Cao, Hongwei Gu**

Key Laboratory of Organic Synthesis of Jiangsu Province, College of Chemistry, Chemical Engineering and Materials Science & Collaborative Innovation Center of Suzhou Nano Science and Technology, Soochow University, Suzhou 215123, P.R. China

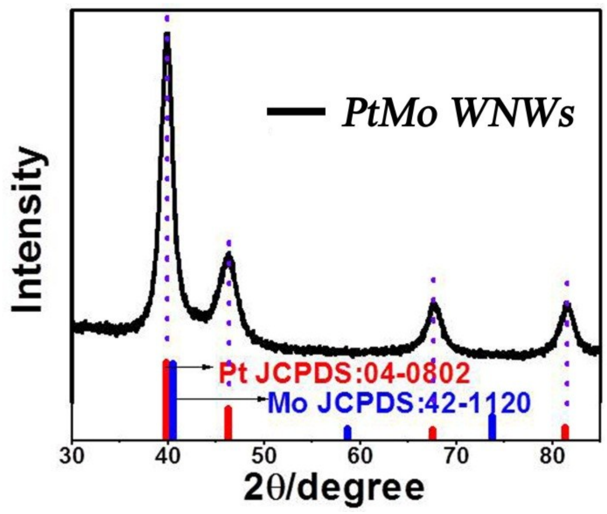

Figure S1. XRD spectra of the PtMo WNWs.

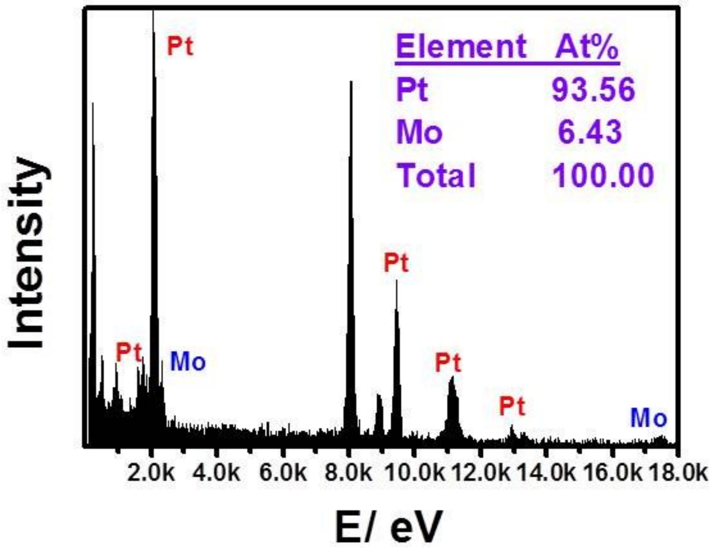

Figure S2. EDS spectra of the PtMo WNWs.

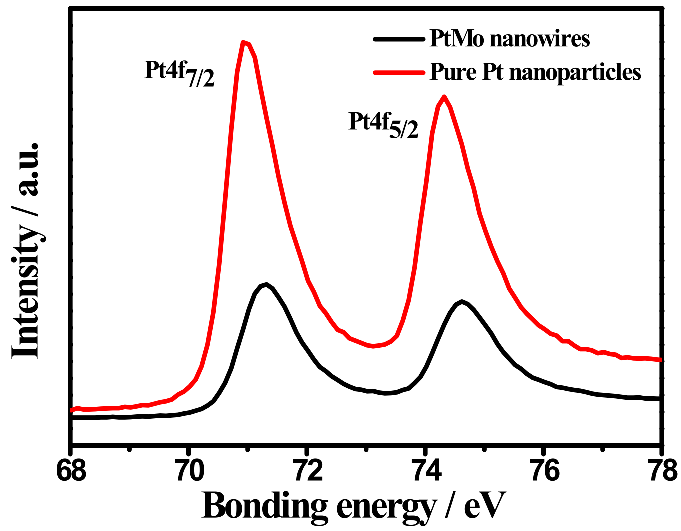

Figure S3. XPS spectra of the PtMo nanowires and Pt nanoparticles.

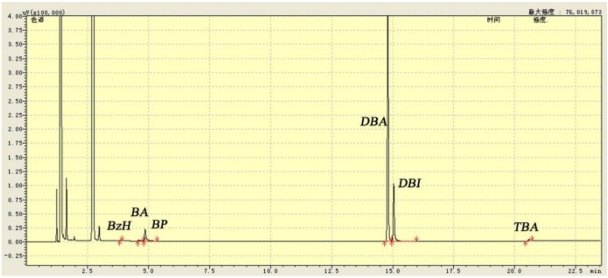

Figure S4. GC spectra of the reductive amination of BzH

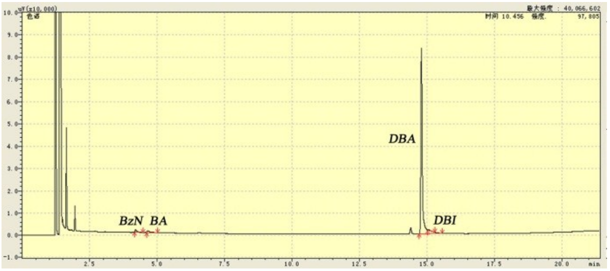

Figure S5. GC spectra of the reductive amination of BzN

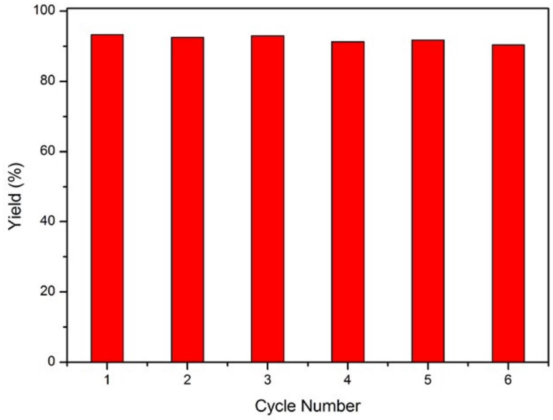

Figure S6. Catalytic stability of Pt nanowires: BzH (1.0 mmol), 25% aqueous ammonia (2 equiv.), *m*-xylene (2 mL), 1 bar H_2, 100°C, 0.5 mol% PtMo WNWs for 24 h.

Table S1. Reductive amination for different molar ratios of BzH to ammonia [a]

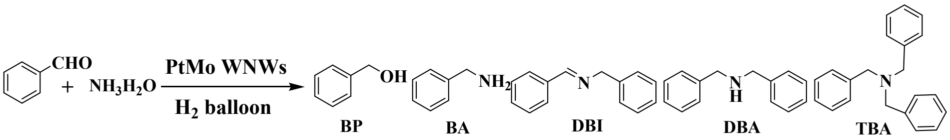

Entry	Ammonia (equiv.)	Conv. (%)[b]	Yield. (%)[b]				
			BA	BP	DBI	DBA	TBA
1	0	99.0	--	99.0	--	--	--
2	0.5	79.9	--	3.8	9.6	62.4	4.1
3	1	100	1.4	--	2.6	94.6	1.5
4	2	100	0.7	0.5	0.8	96.1	0.8
5	5	100	--	18.1	1.2	56.9	23.8

[a] Reaction conditions: BzH (1.0 mmol), and *m*-xylene (2 mL) at 1 bar H_2, 100°C, with 0.5 mol% PtMo WNWs for 24 h. [b] GC yield.

Table S2. Control experiments dealing with the PtMo WNWs catalyzed system [a]

Entry	Catalyst	H_2 (bar)	Conv. (%)[b]	Yield. (%) [c]				
				BA	BP	DBI	DBA	TBA
1	--	1	0	--	--	--	--	--
2	PtMo WNWs	--	0	--	--	--	--	--
3	Pt/C	1	100	--	--	1.7	55.5	42.8
4[b]	Pt/C	1	96.8	--	--	10.1	24.3	52.8

[a] Reaction conditions: BzH (1.0 mmol), and *m*-xylene (2 mL) at 1 bar H_2, 100°C, with 0.5 mol% catalyst for 24 h. [b] BzN (1.0 mmol) and ethanol (2 mL) at 1 bar H_2, 80°C, with 0.5 mol% Pt/C WNWs for 24 h. [c] GC yield.

Table S3. Comparison of the catalytic activities using Pt nanoparticles and Pt nanorods

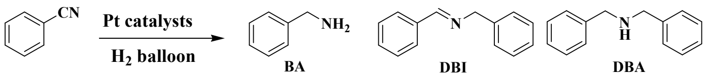

Entry	Catalyst	Conv. (%)	Select. (%) [b]		
			BA	DBA	DBI
1	Pt NPs[1]	88.7	-	74.9	22.0
2	Pt NRs[2]	100	-	89.0	6.0

[1]Wang, C.; Hou, Y. L.; Kim, J.; Sun, S. H. *Angew. Chem. Int. Ed.* **2007**, *46*, 6333.

[2] Kim J.; Lee Y.; Sun S. H. *J. Am. Chem. Soc.*, **2010**, 132, 4996.

NMR analysis for the DBA products:

dibenzylamine: ¹H NMR

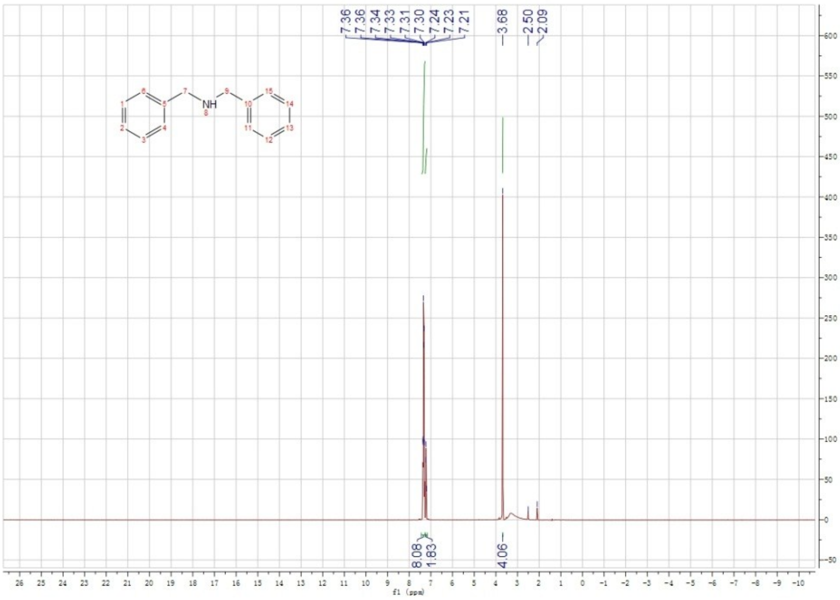

dibenzylamine: ¹³C NMR

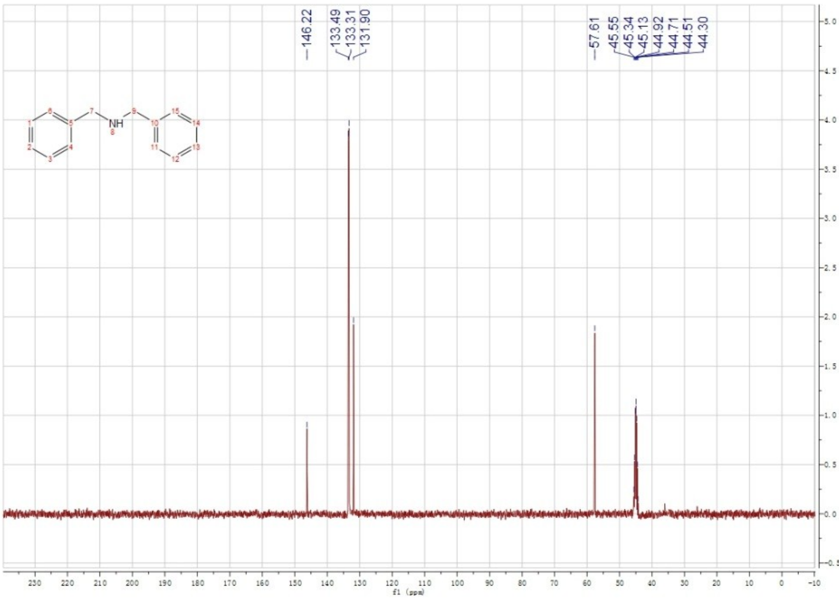

di(2-methyl)benzylamine: ¹H NMR

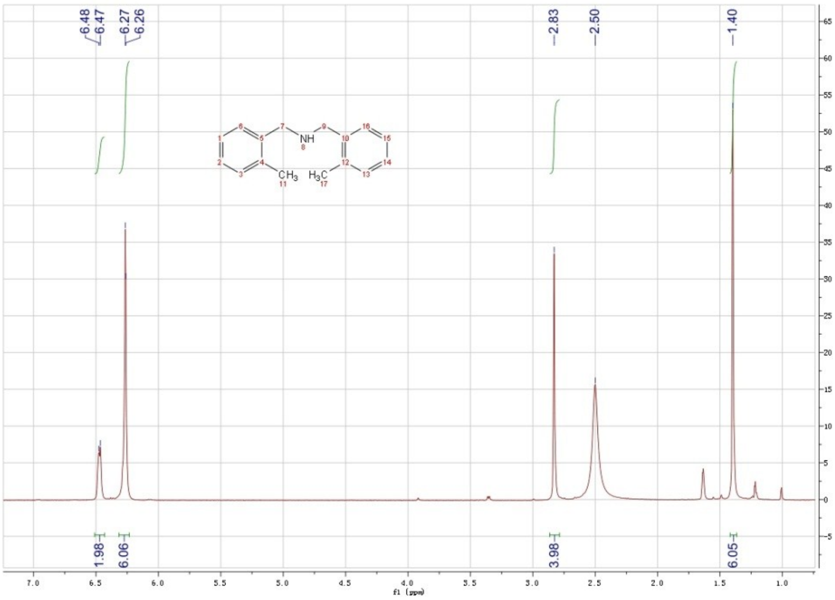

di(2-methyl)benzylamine: ^{13}C NMR

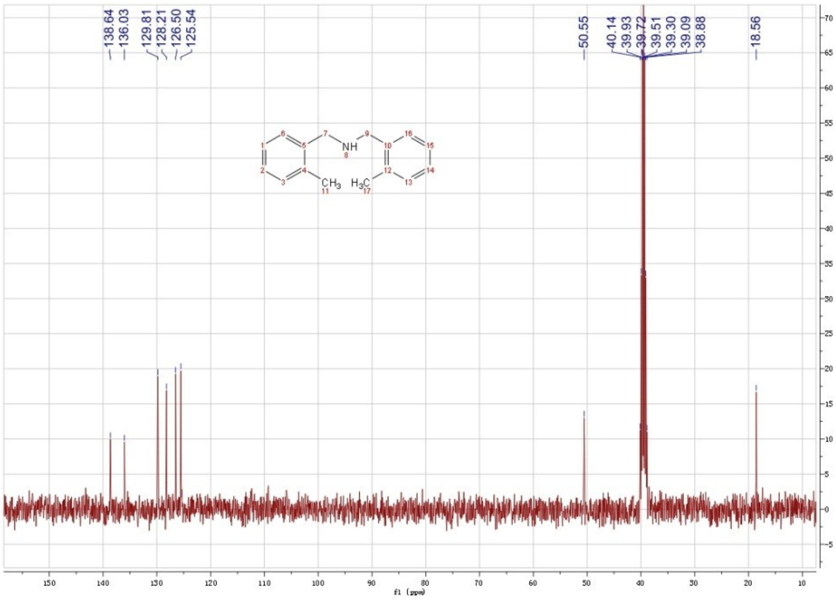

di(3-methyl)benzylamine: ^{1}H NMR

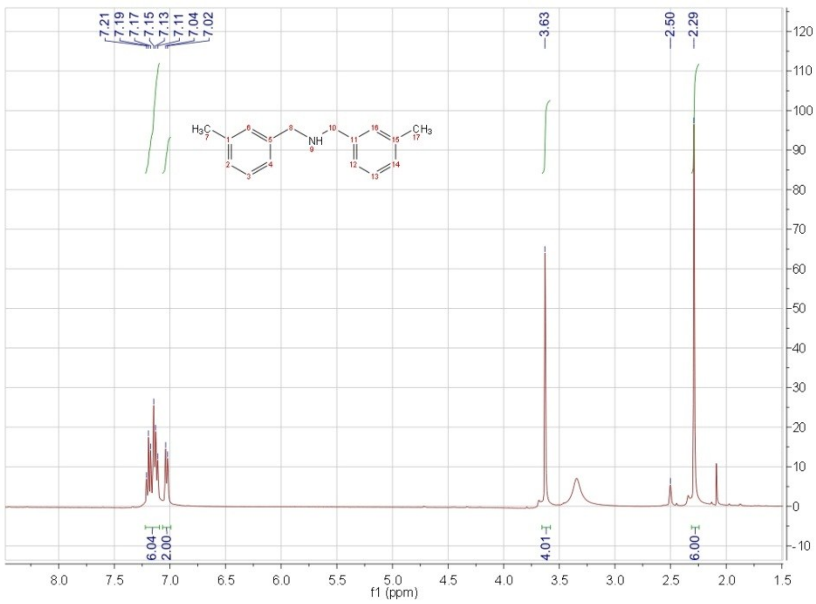

di(3-methyl)benzylamine: ^{13}C NMR

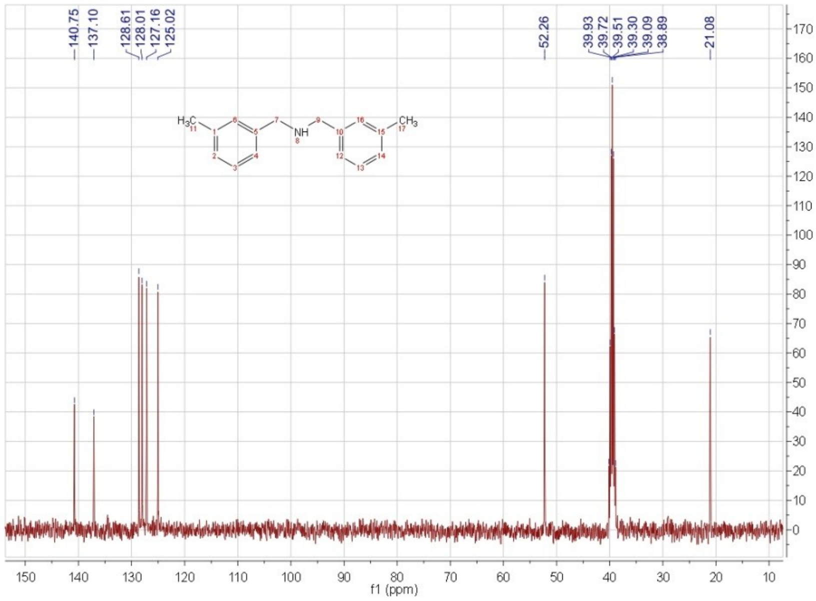

bis(4-methylbenzyl)amine: ^{1}H NMR

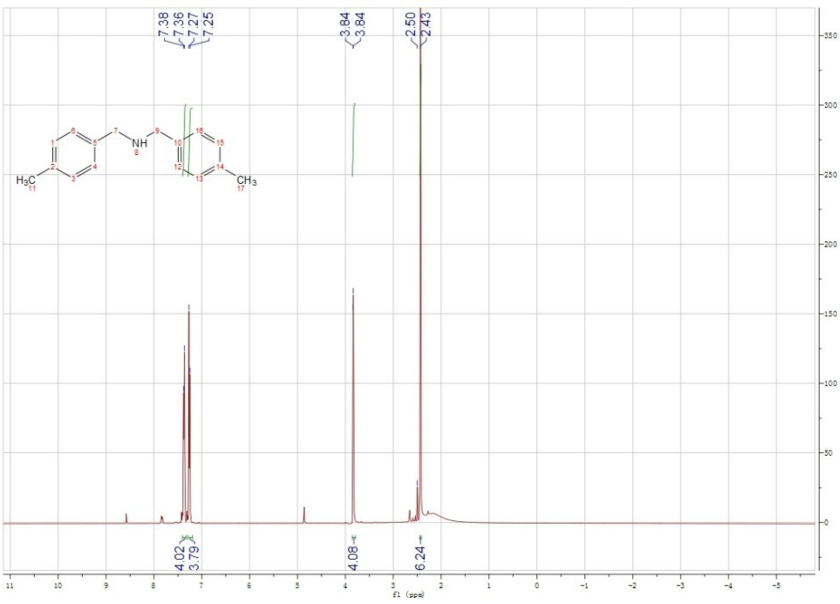

bis(4-methylbenzyl)amine: ^{13}C NMR

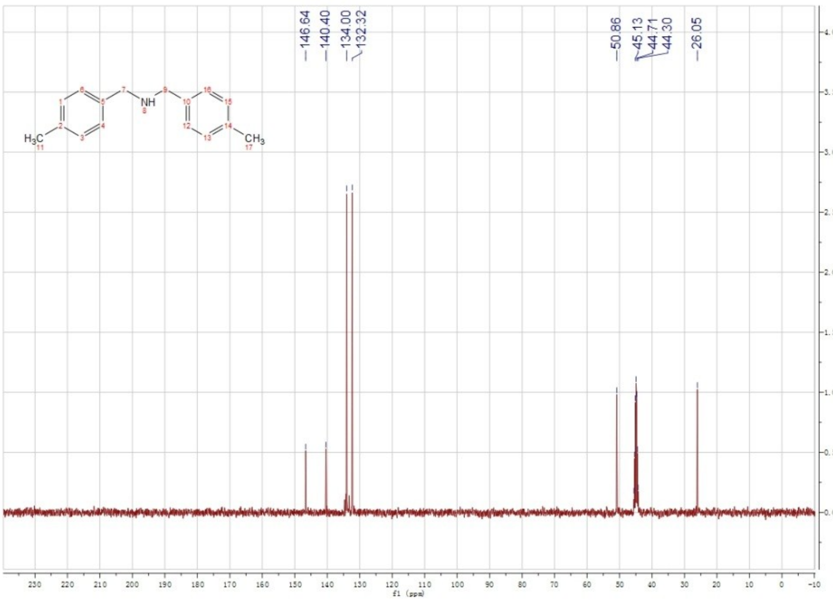

bis(4-methoxybenzyl)amine: ^{1}H NMR

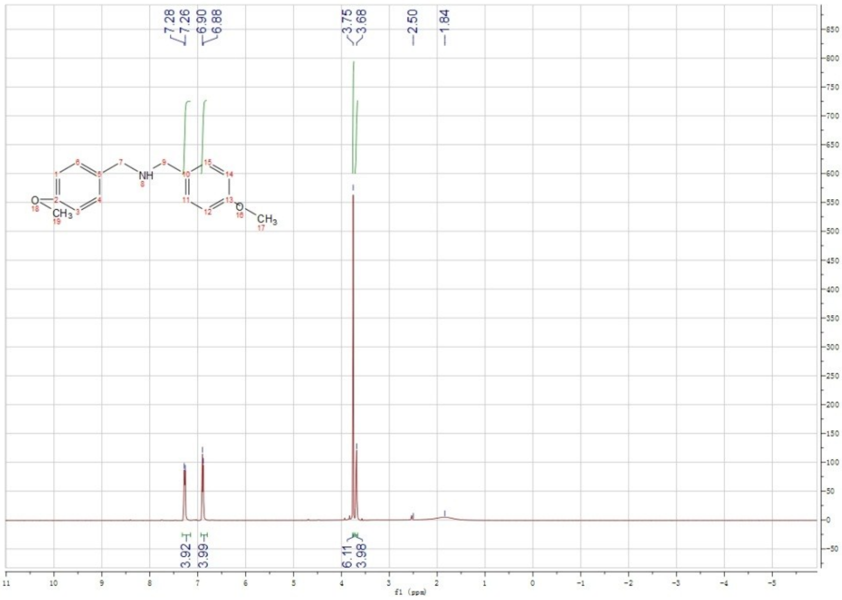

bis(4-methoxybenzyl)amine: ^{13}C NMR

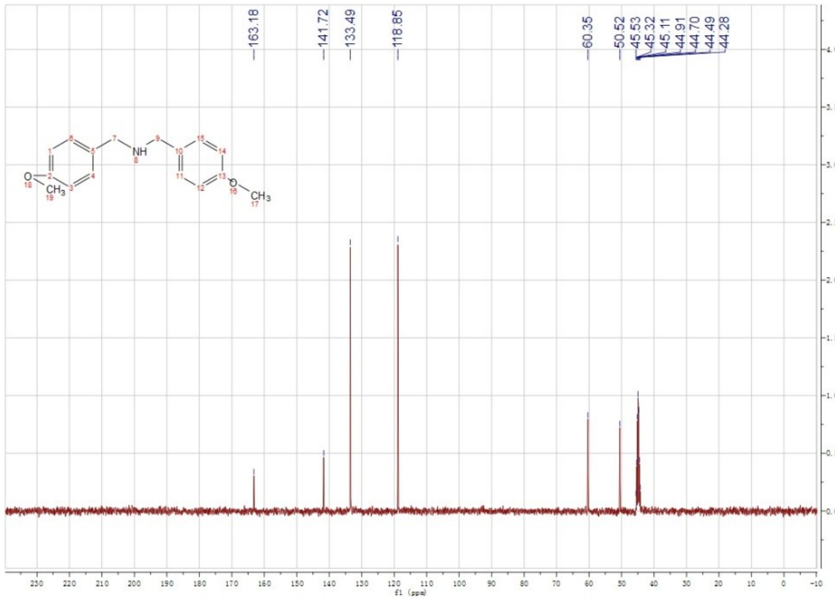

bis(4-chlorobenzyl)amine: ^{1}H NMR

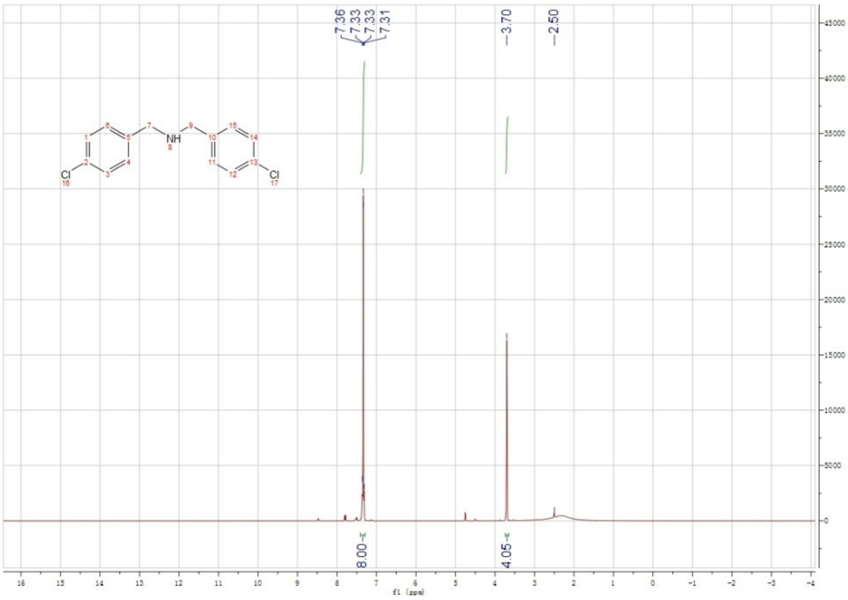

bis(4-chlorobenzyl)amine: ^{13}C NMR

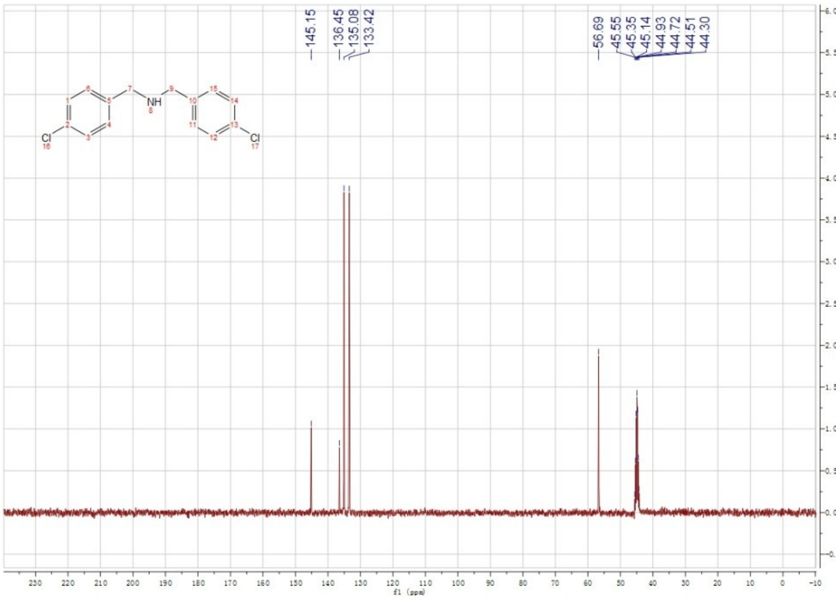

Supplement: RA-008-C8RA00787J-s001 [file RA-008-C8RA00787J-s001.pdf]
